# Porcine Models of Biofilm Infections with Focus on Pathomorphology

**DOI:** 10.3389/fmicb.2017.01961

**Published:** 2017-10-10

**Authors:** Louise K. Jensen, Anne S. B. Johansen, Henrik E. Jensen

**Affiliations:** Section for Experimental Animal Models, Department of Veterinary and Animal Science, Faculty of Health and Medical Science, University of Copenhagen, Frederiksberg, Denmark

**Keywords:** biofilm, pig, animal model, hematogenous osteomyelitis, implant-associated osteomyelitis, chronic wounds, endocarditis, pyelonephritis

## Abstract

Bacterial biofilm formation is one of the main reasons for a negative treatment outcome and a high recurrence rate for many chronic infections in humans. The optimal way to study both the biofilm forming bacteria and the host response simultaneously is by using discriminative, reliable, and reproducible animal models of the infections. In this review, the advantages of *in vivo* studies are compared to *in vitro* studies of biofilm formation in infectious diseases. The pig is the animal of choice when developing and applying large animal models of infectious diseases due to its similarity of anatomy, physiology, and immune system to humans. Furthermore, conventional pigs spontaneously develop many of the same chronic bacterial infections as seen in humans. Therefore, in this review porcine models of five different infectious diseases all associated with biofilm formation and chronicity in humans are described. The infectious diseases are: chronic wounds, endocarditis, pyelonephritis, hematogenous osteomyelitis, and implant-associated osteomyelitis (IAO).

## Introduction

Chronic bacterial infections are a major healthcare problem and of increasing concern due to their high burden with respect to economic costs, increased bacterial antibiotic resistance, high morbidity, and mortality (Archer et al., 2011; Roberts et al., 2015). Biofilm forming bacteria have increased tolerance toward antimicrobials as well as mechanical removal and are one of the reasons why chronic bacterial infections are difficult to treat (Costerton et al., 1999; Stewart, 2014). In order to study bacterial biofilms in chronic infections, it is important to have discriminative and reproducible animal models (Costerton et al., 1999; Bjarnsholt, 2013). The pig resembles humans anatomically, physiologically and immunologically. Furthermore, conventional pigs spontaneously (naturally occurring infections) develop many of the same chronic bacterial infections as seen in humans (Harris and Alexander, 1999). Therefore, it is possible to induce the disease experimentally in a discriminative way making the pig a reliable model for the study of biofilm related infectious diseases. This is not the case for many spontaneous infections in rodents, which do not adequately represent features of human disorders. In general, the use of pigs as experimental animals is intensely increasing and it is reasonable to expect that more porcine models of bacterial biofilm infections will be developed in the future. Therefore, the aim of the present paper is to review and describe existing porcine models of bacterial biofilm infections in humans with special focus on pathomorphology.

### Biofilm

Biofilm was described by Burmølle et al. (2010) as: “A coherent cluster of bacterial cells imbedded in a biopolymer matrix, which, compared with planktonic cells, shows increased tolerance to antimicrobials and resists the antimicrobial properties of the host defense” (Burmølle et al., 2010).

In other descriptions of biofilm it is stated that either an abiotic or a biotic surface must be present for biofilm to form (Costerton et al., 1995, 1999; Davey and O'toole, 2000; Flemming and Wingender, 2010). It has however, also been suggested that biofilm does not need a surface in order to be established, as bacteria may attach to each other and form biofilm (Donlan and Costerton, 2002; Archer et al., 2011; Bjarnsholt, 2013). Another important characteristic of biofilm, is that the bacteria change phenotypic expression in regard to growth, gene expression and protein synthesis (Costerton et al., 1995, 1999, 2003; Davey and O'toole, 2000; Brady et al., 2008; Burmølle et al., 2010). In a study by Costerton et al. (2003), it was observed that all chronic bacterial diseases examined during a 12 year period, contained biofilm (Costerton et al., 2003). Moreover, it has been recommended that all refractory chronic bacterial diseases should be analyzed for the presence of biofilm (Donlan and Costerton, 2002).

Bacteria form biofilm in a number of situations: (1) it can be a defense mechanism when bacteria are in a hostile environment, (2) it can serve as a favorable habitat, if the bacteria are in an environment with a low amount of nutrients and (3) it can result from a mutation or a so called default mode of the bacteria (Bjarnsholt, 2013).

The initiation of biofilm formation is usually mediated by flagella and/or pili (Costerton et al., 1999). After adhering to other bacteria and/or a surface, a monolayer of bacteria is formed and develops into micro-colonies (Costerton et al., 1999). Following formation of micro-colonies the extracellular matrix is formed (Costerton et al., 1999). The matrix, which can be formed within 48 h after infection, is usually produced by the bacteria (Davis et al., 2007; Bjarnsholt, 2013). However, sometimes host components are also embedded within biofilm (Bjarnsholt, 2013; Stewart, 2014). The matrix consists of several components; proteins, lipids, extracellular DNA, polysaccharides, and other bacterial macromolecular components. Moreover, it has been realized that different bacteria are embedded in different components of biofilm matrix (Bjarnsholt, 2013).

An increased tolerance of biofilm forming bacteria is seen toward antimicrobials which may be due to a number of factors. Matrix may possess the ability to bind and inactivate antibacterial agents (Bjarnsholt, 2013). Furthermore, bacteria growing as a biofilm are less metabolically active, due to the decreased amounts of nutrients available, making them more tolerant to several antimicrobials (Bjarnsholt, 2013). In addition, bacteria in biofilm also show increased tolerance toward the host's immune system (Bjarnsholt, 2013; Stewart, 2014). The mechanism behind this has not been fully elucidated, but it appears that the presence of biofilm constantly stimulates polymorphonuclear leucocytes (PMNs). PMNs are, however, not able to phagocytize biofilm, presumably due to the size of the biofilm, leading to the phenomenon “frustrated phagocytosis” (Bjarnsholt, 2013; Stewart, 2014). The role of the adaptive immune response has not been fully clarified with respect to biofilm. Although, in *Staphylococcus aureus* biofilm it has been realized that several factors inhibits the activation and effects of the adaptive immune response (Kim et al., 2012).

**Figure 1:**
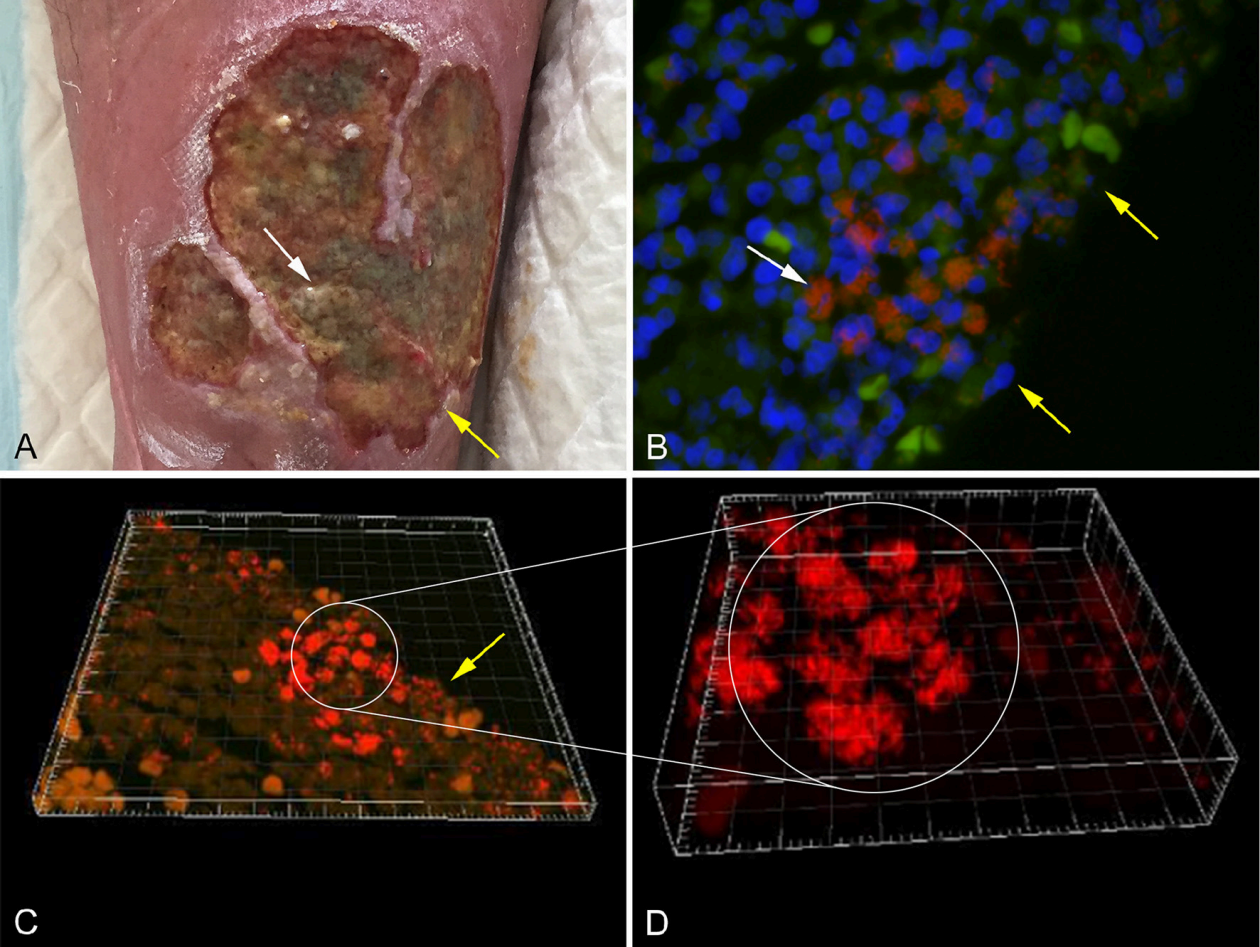
**(A)** Chronic venous leg ulcer. **(B)** Biofilm of *P. aeruginosa* [red stain] and *S. aureus* [green stain], identified by specific PNA FISH probes, surrounded by host cells (DAPI [blue stain]) in a human chronic wound. **(C)** CSLM three dimensional imaging of picture B. **(D)** Enlargement of picture C. The white arrows point to bacterial aggregates and the yellow arrows point to the wound surface (Kirketerp-Møller et al., 2008).

Several methods have been applied in order to demonstrate biofilm formation and function, in relation to different infectious diseases (Lebeaux et al., 2013). It has been proven difficult to culture biofilm embedded bacteria and some samples may need ultra-sonication before cultivation (Burmølle et al., 2010). Other methods used to identify biofilm embedded bacteria include peptide nucleic acid fluorescence *in situ* hybridization (PNA FISH; Figure 1B), electron microscopy, immunohistochemistry staining, and confocal scanning laser microscopy (CSLM; Costerton et al., 1999; Bjarnsholt, 2013). CSLM is becoming the method of choice, as it enables analysis of a fully hydrated and living biofilm *in situ* (Figures 1C,D; Costerton et al., 1995; Kirketerp-Møller et al., 2008).

### Biofilm *in Vitro* and *in Vivo*

Several approaches have been applied to study the complexity of bacterial biofilms. *In vitro* experiments were used in the early studies of biofilm and much knowledge about biofilm physiology and formation has been achieved using *in vitro* models (Bjarnsholt et al., 2013; Lebeaux et al., 2013). However, *in vitro* and also *ex-vivo* (Yang et al., 2017) studies have to be supplemented with *in vivo* studies if the response of the immune system toward bacterial biofilm infections is investigated (Rumbaugh and Carty, 2011; Roberts et al., 2015). This was also recently demonstrated with references to porcine infections due to *Actinobacillus pleuropneumoniea* (Tremblay et al., 2017).

Biofilm formation *in vitro* and *in vivo* is significantly different with respect to a number of characteristics (Bjarnsholt et al., 2013). *In vitro* biofilm of *Pseudomonas aeruginosa* forms “mushroom” like structures (Bjarnsholt, 2013; Ghanbari et al., 2016), which are not observed *in vivo* (Bjarnsholt et al., 2013). Another important difference is that *in vitro* biofilm is solely made up by bacterial derived components, whereas *in vivo* biofilm contains a mixture of bacterial and host derived elements (Bjarnsholt, 2013; Stewart, 2014). The size of biofilm formations is also different *in vitro* compared to *in vivo*. *In vivo* biofilms have a maximum diameter of 200 μm, whereas *in vitro* biofilm can reach up to several centimeters (Bjarnsholt et al., 2013). The limitation in size has been suggested to be related to oxygen depletion in the local environment (Roberts et al., 2015). Finally, the infectious biofilm formation *in vivo*, also enables the study of the host immune response toward biofilm (Coenye and Nelis, 2010). The pros and cons for *in vitro* and *in vivo* studies of bacterial biofilms are shown in Table 1 (Lebeaux et al., 2013).

**Table 1:** Pros and cons of studying biofilm in regard to bacterial diseases *in Vitro* vs. *in Vivo*.

	**Pros**	**Cons**
***In vitro***	Simple	No host influence
	Reproducible	Inferior in treatment studies
	Cheap	“Mushroom” structure
	Non-invasive	
***In vivo***	Host influence	Expensive
	Ideal in treatment studies	Complex
	Natural biofilm formation	Biological variation
		Invasive

## Comparative anatomy, physiology, and immunology of pigs and humans

**Figure 2:**
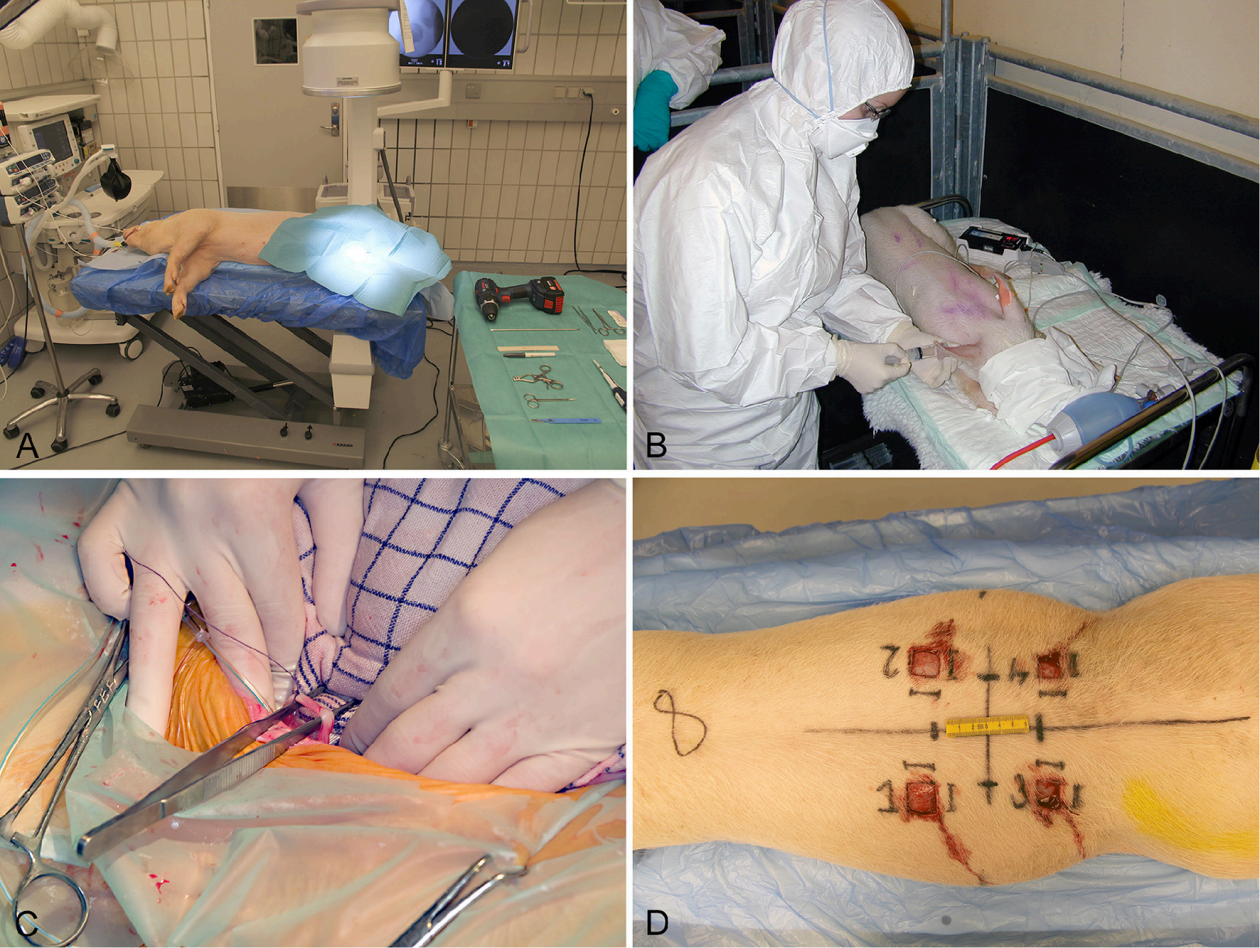
Establishment of four different models in conventional pigs of 30 kg. **(A)** Implant associated osteomyelitis, a drill hole is created in the right tibia followed by injection of bacteria and insertion of a small metal implant (Jensen et al., 2017). **(B)** Intravenous inoculation of bacteria for induction of endocarditis. Four days prior to inoculation, a permanent catheter was placed in the left ventricle (Christiansen et al., 2013b). **(C)** Free dissection of the right ureter, followed by insertion of a catheter used for inoculation of bacteria directly in the renal pelvis (Isling et al., 2011). **(D)** Four wounds created on the back at different time intervals for bacterial inoculation.

**Figure 3:**
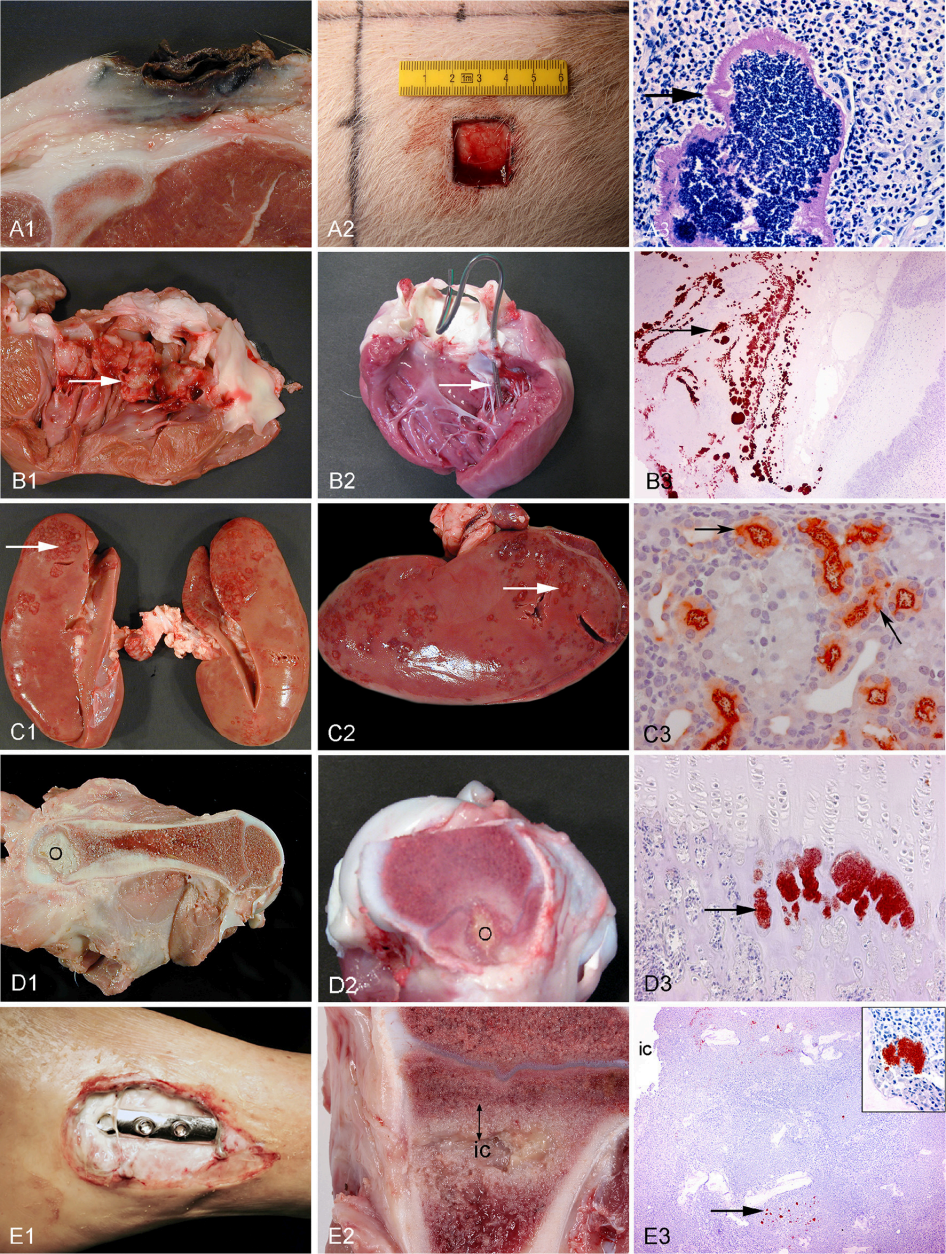
Left column: chronic spontaneous bacterial infections in conventional pigs **(A–D)** and a human **(E)**. Middle column: experimental porcine models of human infections. Right column (except from picture A3): microscopy of the lesions present in the middle column. Row A: Chronic wounds. **A1**: Shoulder ulceration. **A2**: wound located on the back. **A3**: Bacteria (arrow) in a shoulder ulceration from a conventional pig. Row B: Endocarditis. **B1**: Left side, thrombotic valvular endocarditis (arrow). **B2**: A permanent catheter (arrow) inserted into the left ventricle prior to inoculation of bacteria. **B3**: Immunohistochemical staining of *S. aureus* (arrow) on the mitral valve (Christiansen et al., 2013b). Row C: Pyelonephritis. **C1, C2**: Polar located lesions of pyelonephritis (arrows). **C3**: Immunohistochemical staining of *E. coli* (arrows) in the proximal tubuli (Isling et al., 2011). Row D: Hematogenous osteomyelitis. **D1, D2**: Purulent osteomyelitis (O) in the femur. **D3**: Immunohistochemical staining of *S. aureus* (arrow) located in the capillary loops of the metaphysis (Johansen et al., 2012b). Row E: Implant-associated osteomyelitis. **E1**: Infected osteo-syntheses of the ankle. **E2**: Peri-implant infected bone tissue (double arrow) surrounding the implant cavity (ic), the implant has been removed. **E3**: Immunohistochemical staining of *S. aureus* (arrow) and in the insert (Jensen et al., 2017).

Several different animal models have been applied in order to study biofilm *in vivo*. In relation to chronic bacterial diseases, mice, rats, and rabbits have been used most frequently (Rumbaugh and Carty, 2011). Only a few large animal models using pigs, sheep, goats, monkeys, and dogs have been developed (Rumbaugh and Carty, 2011). In recent years, the use of pigs as experimental animals has increased. Pigs have especially been applied in studies of toxicity, metabolism, cancer, dermatology, cardiology, and neurology (Swindle et al., 2012). However, during the last ten years several porcine models of bacterial diseases have been developed (Isling et al., 2011; Meurens et al., 2012; Christiansen et al., 2013b; Jensen et al., 2017). Figures 2, 3 shows the diversity of the pig as a model for human bacterial diseases. An advantage of using pigs as a model for bacterial infections is their immune response, which is quite comparable to that of humans (Meurens et al., 2012). In a study comparing the porcine, and human genome it was found that there is a 78% similarity, both structurally and functionally, between human and porcine immune related proteins (Dawson, 2011). The population of immune cells in humans and pigs is also alike. As in humans, pigs have a large percentage of PMNs in the peripheral blood (Meurens et al., 2012).

Porcine models can be based on conventional pigs or mini-pigs of different breeds. The growth rate of conventional pigs is high (1 kg at birth, 100 kg at 4 months and the body weight of an adult is >200 kg) compared to mini-pigs (0.5 kg at birth, 12–14 kg at 4 months and the body weight of an adult is 40–80 kg), making mini-pigs a more favorable model for adults (Swindle et al., 2012). Pigs are available as outbred and inbred. Using inbred pigs will allow a more uniform outcome of studies, in which the outbred pigs will allow the impact of biological diversity, just as in the human population (Meurens et al., 2012). Furthermore, the health status and full pedigree can usually be acquired for both conventional pigs and mini-pigs (Meurens et al., 2012). Min-pigs are available from Sinclair Bioresource (Hanford, Sinclair, Yucatan, Yucatan Micro) and Ellegaard Göttingen Minipigs (Göttingen Minipigs). Conventional pigs are supplied by conventional farmers.

## Porcine models of chronic bacterial infectious diseases

Pigs have been used to model the following chronic bacterial diseases, all known to be associated with biofilm formation in humans; chronic wounds, endocarditis, pyelonephritis, hematogenous osteomyelitis, and implant-associated osteomyelitis (IAO). In this review, these models will be described and the advantages of using pigs as a model for the five diseases will be elucidated with regard to how pigs are comparable to human's skin, heart, kidneys, and bones.

The inclusion and exclusion criteria for this review: The keywords “porcine model,” “biofilm,” “chronic wounds,” “endocarditis,” “pyelonephritis,” “hematogenous osteomyelitis,” “implant-associated osteomyelitis” were searched for in different combinations. The primary databases used were Google Scholar, REX, Web of Science and PubMed. The criteria which the studies had to fulfill in order to be enclosed in this review were as follows;

1. It had to be a study of chronic wounds, endocarditis, pyelonephritis, hematogenous osteomyelitis, or implant-associated osteomyelitis.
2. The study had to use a porcine model.
3. The pigs had to be inoculated with bacteria in order to develop infection.

## Porcine models of chronic wounds

Pigs are commonly applied in experimental wound studies (Figure 3A2). The skin of pigs is comparable to that of humans in a number of ways. Pig skin has little hair and is well attached to the subcutaneous layer, as in humans (Swindle and Smith, 1998). Wound healing in pigs has been found to be similar to that of humans (Sullivan et al., 2001). The major differences are the cutaneous blood supply as well as the thickness of the skin (Swindle and Smith, 1998; Liu et al., 2010). Although the thickness of the skin is greater in pigs, the ratio between dermis and epidermis is similar to that of humans (Meurens et al., 2012). The subcutis in pigs is divided into three different layers, which are all separated by fascia (Driskell et al., 2014). In humans, the subcutis is only divided into two layers (Driskell et al., 2014). Finally, the sweat glands in porcine skin are all apocrine, whereas in humans eccrine sweat glands are dominating (Liu et al., 2010).

The pig is suitable as a model for chronic wounds due to its similarity to humans with respect to the structure of the skin, but also because chronic wounds regularly are found in conventional pigs. Accidentally occurring chronic wounds may be found all over the skin of pigs; however, In recent years focus has been on shoulder ulcerations in sows (Figure 3A1). Shoulder ulcerations in pigs are caused by pressure and the pathogenesis is a progression of damage from the top and down through the dermal layers (Maxie, 2007; Jensen, 2009; Schomberg et al., 2016). This pathogenesis is also seen in humans, however, a “reversed” pathogenesis, in which the progression can be from the inside and out is also seen in humans (Anderson, 1976). A major difference in the pathogenesis of human pressure ulceration is that they can be complicated by osteitis/osteomyelitis, which has not been associated with pressure ulcers in pigs (Jensen, 2009). Another important difference between pressure ulcers in humans and pigs is that in pigs, they occur in otherwise healthy individuals, whereas in humans they usually develop due to an underlying disease (Dahl-Pedersen et al., 2013). In humans, the most important pathogen in chronic wounds is *S. aureus* (Bowler, 2002), whereas *Trueperella pyogenes* is the most common pathogen in shoulder ulcerations of sows (Lund, 2003; Dahl-Pedersen et al., 2013). Along with pressure ulcers, there are two other types of chronic wounds, which dominate in humans; diabetic ulcers and venous ulcers (Figure 1A; Medina et al., 2005). Although diabetic and venous ulcers do not occur in pigs under natural circumstances, the pig has been used as a model for diabetic wounds (Seaton et al., 2015). Porcine models of burn wounds and hypertrophic scars have also been developed (Seaton et al., 2015). In 2003 Breuing et al. established a partial thickness burn wound model with *S. aureus* as inoculum (Table 2). They used a chamber to cover the burn wound, by which it was possible to study the wounds continuously (Breuing et al., 2003). Davis et al. (2007) created a partial thickness wound using an electrokeratome, which was also inoculated with *S. aureus*. By using electron microscopy and epifluorescence microscopy (Table 2), they demonstrated the formation of biofilm after 48 h. Furthermore, they also showed that biofilm could not be eradicated with the antibiotics tested (Davis et al., 2007). A novel full thickness diabetic wound model developed by Hirsch et al. (2008) showed that diabetic pigs maintained a significant infection compared to the non-diabetic pigs (Table 2). Furthermore, a significant delay in wound healing was found in the diabetic wounds (Hirsch et al., 2008). A full thickness wound model by Roche et al. (2012; Table 2), showed that biofilm formation resulted in delayed healing (Roche et al., 2012). Nusbaum et al. (2012) inoculated *S. aureus* into a deep dermal wound model (Table 2) in order to study the effect of different types of wound debridement: plasma-mediated bipolar radiofrequency ablation, hydrosurgery system, and sharp debridement. They achieved a significant reduction of bacteria in all debridement groups and a significant reduction of *S. aureus* in the plasma-mediated bipolar radiofrequency ablation groups (Nusbaum et al., 2012). Finally, a full thickness porcine wound model was established using *P. aeruginosa* as inoculum (Table 2). In the model, the effect of different therapeutical strategies using negative pressure wound therapy was evaluated (Davis et al., 2013).

**Table 2:** Porcine models of chronic wounds.

**References (year)**	**Model design**								**Inoculum**				**Outcome**	
	**Number of animals**	**Age (weeks)**	**Weight (kg)**	**Sex**	**Breed**	**Number of wounds**	**Infection time**	**Implant/modification**	**Bacteria**	**Dose CFU/ml**	**Volume**	**Route**	**Bacterial verification T/I**	**Infection stage**
Breuing et al., 2003	3	24	45	F	Yorkshire	48	2–47 days	Partial thickness (burn) wounds Wound chamber	*S. aureus* MRSA 25923	10^8^	1.2 ml	Topical	T: CFU + Histology I: NA	Acute
Davis et al., 2007	6	NR	25–35	F	NR	<279	48 h	Partial thickness wounds Wound dressing	*S. aureus* ATCC 6,538	10^7^	NR	Topical	T: Histology + SEM + EpiM I: NA	Acute
Hirsch et al., 2008	4	NR	50–60	F	Yorkshire (±diabetic)	56	12 days	Full thickness (diabetic) wounds Wound chamber	*S. aureus* MRSA 25923	2 × 10^8^	NR	Topical	T: CFU + Histology I: NA	Chronic
Roche et al., 2012	40	NR	20–25	F	Yorkshire crossbred	640–800	21 days	Full thickness wounds Wound dressing	*S. aureus* MRSA ATCC 33592+PJR006	1 × 10^7^ 1 × 10^8^	0.5 or 2 ml	Topical	T: CFU + Histology + SEM I: NA	Chronic
Nusbaum et al., 2012	9	NR	35–40	F	NR	135	23 days	Partial thickness Wound dressing	*S. aureus* MRSA USA300	10^6^	25 μl	Topical	T: CFU + Histology I: NA	Chronic
Davis et al., 2013	6	NR	40–55	F	NR	36	21 days	Full thickness wounds Wound dressing	*P. aeruginosa* 6538	500	NR	Topical	T: PCR + CFU I: NA	Chronic

*T, verification method in tissue; I, verification method on implant; CFU, colony forming units; SEM, scanning electron microscopy; EpiM, epifluorescence microscopy; NR, not registered; F, female. NA, not available*.

## Porcine models of endocarditis

Due to porcine similarities in the cardiovascular system, pigs have been used as a model in a number of cardiac studies, e.g., transplantation, experimental atherosclerosis, and endocarditis (Swindle et al., 2012; Schomberg et al., 2016). The hemodynamics of the porcine cardiovascular system is similar to humans, however, there are differences between different breeds and ages of pigs (Swindle and Smith, 1998; Swindle et al., 2012). In contrast to humans, pigs have a large left azygos (hemiazygos) vein, which enters into the coronary sinus, instead of the superior vena cava (Swindle and Smith, 1998; Swindle et al., 2012). Another difference is that the semi-lunar valves in pigs are slightly smaller compared to that of humans (Ibrahim et al., 2006). The hearts of pigs come in many different sizes, however, the heart of a mini-pig is equivalent to about 0.3–0.5% of the total bodyweight, thus, a mini-pig of about 40–50 kg, has a heart of similar size as an adult human (Swindle and Smith, 1998).

Endocarditis occurs spontaneously in conventional pigs, and has been characterized, both histopathologically and microbiologically (Jensen et al., 2010a). In pigs, the lesions of endocarditis are mainly located on the mitral valves, sometimes with secondary lesions in the adjacent mural wall (Figure 3B1). Histopathologically, the vegetation is generally made up of granulation tissue, surrounded by fibrin. However, vegetation made up of granulomatous inflammation with mineralization may be seen, particularly in *Streptococcus* infections (Jensen et al., 2010a). Others have described the vegetation found in porcine endocarditis as “Cauliflower-like” (Geissinger et al., 1973). In humans, the appearance of the lesion is similar to that of pigs. The lesions can be quite large and are also made up by fibrin, and the embedded bacteria are surrounded by leukocytes and granulation tissue (Anderson, 1976). The most common infectious agents of porcine endocarditis are *S. suis* and *Erysipelothrix rhusiopathiae* (Jensen et al., 2010a). In contrast, the most important pathogen in humans is *S. aureus* (Murdoch et al., 2009). The pathogenesis in pigs has not been established, however, it is believed that there has to be a persistent or recurrent bacteremia (Maxie, 2007). In humans, infectious endocarditis mainly develops secondary to a state of non-bacterial thrombotic endocarditis (Christiansen et al., 2013b).

The first porcine model of endocarditis was described by Jones (1969) who established a non-traumatic endocarditis model, in which four different strains of *Streptococci* were inoculated intravenously. The strains were from Lancefield group C and L, respectively. Strain S85 (Group L), resulted in endocarditis in the pigs and was used in his further studies (Table 3; Jones, 1969, 1981, 1982). He found that macroscopic lesions developed as early as 18 h after inoculation and that the lesions in general matched those seen in humans (Jones, 1969). Geissinger et al. (1973) did a study using both conventional and gnotobiotic pigs, where they inoculated the bacteria sub-cutaneously. In that study, they also used two different strains of *S. aureus* (A and B) as well as one strain of *E. rhusiopathiae*. The study showed that strain A of *S. aureus*, was the only one which resulted in endocarditis in both conventional and gnotobiotic pigs (Geissinger et al., 1973). In another study by Jones (1981), he examined the lesions 3–14 days after inoculation, using the same experimental procedure as in 1969 (Jones, 1981). Finally, Jones examined the development of lesions 18–48 h after inoculation. The lesions were again macroscopically visible 18 h after inoculation (Jones, 1982). In 1986, Johnson et al. tried to reproduce the findings of Jones (Jones, 1969, 1981, 1982), however, only 11% of the pigs developed endocarditis (Johnson et al., 1986). Interestingly, insertion of a non-permanent catheter through the carotid artery and into the left ventricle followed by intravenous inoculation of *Streptococci* (Group C) resulted in endocarditis in 94% of the pigs (Johnson et al., 1986). Another study using catheterization was done by Dewar et al. (1987) who used mini-pigs and inoculations of *S. sanguis* (Group H), which resulted in the development of endocarditis in 75% of the pigs (Dewar et al., 1987). Recently, two studies were carried out by Christiansen et al. (2013a,b). In these studies a permanent catheter was also placed through the carotid artery and into the left side of the heart. The bacteria inoculated were two strains of *S. aureus* which were isolated from a pig and a human, respectively. The porcine strain produced endocarditis in both studies, using an inoculum dose of 10^7^ CFU/ml, whereas infection by the human strain failed to induce endocarditis (Figures 3B2,B3; Christiansen et al., 2013a,b).

**Table 3:** Porcine models of endocarditis.

**References (year)**	**Model design**							**Inoculum**				**Outcome**		
	**Number of animals**	**Age (weeks)**	**Weight (kg)**	**Sex**	**Breed**	**Infection time**	**Implant/modification**	**Bacteria**	**Dose CFU/ml**	**Volume**	**Route**	**Bacterial verification T/I**	**Infection stage**	Success rate^*^
Jones, 1969	17	8–12 and 12–14	NR	M/F	Yorkshire	2–47 days	None	*β-haemolytic Streptococci* S21 and S57 (Group C) S34 and S85 (Group L)	10^8^	10 ml	I.V.	T: Microbiology + Histology I: NRV	Acute	S85: 77%
Geissinger et al., 1973	64	12–20 and 3 or 10	NR	M/F	Yorkshire Conventional and Gnotobiotic	2–12 days	None	Two strains of *S. aureus* A and B *E. rhusiopathiae* P2-8	A: 2 × 10^9^–9 × 10^10^ B: 8 × 10^11^ *E. rhusiopathiae:* 4 × 10^10^	7-10 ml	S.C.	T: Histology (LM and SEM) I: NRV	Acute	Strain A: Conventional: 37% Gnotobiotic: 55%
Jones, 1981	60	8–12	NR	M/F	Yorkshire	10–35 days	None	*β-haemolytic Streptococci* S85 (Group L)	10^8^	10 ml	I.V.	T: Microbiology I: NRV	Acute	50%
Jones, 1982	23	8–12	NR	M/F	Yorkshire	18–48 h	None	*β-haemolytic Streptococci* S85 (Group L)	10^8^	10 ml	I.V.	T: Microbiology + Histology I: NRV	Acute	60%
Johnson et al., 1986	41	3–4	NR	M/F	Crossbred	24 days	Catheter	*β-haemolytic Streptococci* (Group C)	1 × 10^7^	NR	I.V.	T: Microbiology + Histology I: NR	Acute	Catheter: 94% No catheter: 11%
Dewar et al., 1987	10	NR	25–35	M	Göttingen mini-pigs	20–55 days	Catheter	*S. sanguis* NCTC7864	3 × 10^8^	NR	I.V.	T: NR I: NR	NR	75%
Christiansen et al., 2013b	14	6–9	13–25	F	Yorkshire-Landrace crossbred	1–11 days	Catheter	*S. aureus* S54F9 and NCTC8325-4	10^5^–10^7^	13-25 ml	I.V.	T: Microbiology + Histology I: NR	Acute	Both strains: 25% S54F9 10^7^ CFU: 100%
Christiansen et al., 2013a	17	6–10	13–25	F	Yorkshire-Landrace crossbred	1–11 days	Catheter	*S. aureus* S54F9 and NCTC8325-4	10^5^–10^8^	13-25 ml	I.V.	T: Microbiology + Histology I: NR	NR	Both strains: 22% S54F9 10^7^ CFU: 100%

* *The percentage of animals with infection. T, verification method in tissue; I, verification method on implant; CFU, colony forming units; NR, not registered; F, female; M, male; I.V, intravenous; S.C., sub cutaneous; NRV, not relevant*.

## Porcine models of pyelonephritis

The kidneys of pigs are anatomically quite similar to those of humans as both have multirenculate and multipapillate kidneys. Another similarity between pigs and humans is the glomerular filtration rate (Ibrahim et al., 2006). An important difference is related to the vascularity of the kidneys. In pigs the vascular plane is parted transversely and not longitudinally as in humans (Swindle et al., 2012). The similarities have allowed the development of vesicourethral and intrarenal reflux models in pigs (Swindle and Smith, 1998).

In humans, the chronic form of pyelonephritis is usually caused by urinary reflux and urethral obstruction (Damjanov and Linder, 1996). The most common pathogens involved are *Escherichia coli* and *Proteus* sp. Pathologically, fibrotic scarring is present at the poles, due to urinary reflux as well as dilated calices. Histopathologically, interstitial fibrosis is seen along with mononuclear leukocyte infiltration and tubular atrophy (Damjanov and Linder, 1996). The lesions are asymmetrical which results in an irregular contraction of the kidneys. In pigs, the lesions are similar to those in humans and the most common pathogen is also *E. coli* (Figure 3C1). However, other pathogens such as *Staphylococcus, Streptococcus, Enterobacter, Proteus*, and *Actinobaculum* have also been isolated from cases of porcine pyelonephritis (Maxie, 2007).

Only a few porcine models of pyelonephritis have been created. A vesico-urethral reflux model was established in Sinclair mini-pigs for the study of chronic atrophic pyelonephritis (Table 4; Hodson et al., 1975). Both the inoculated and non-inoculated group developed scarring composed of fibrosis and leukocyte infiltration. Six of the inoculated pigs in the experiment eradicated the induced infection with *E. coli*, spontaneously, whereas 11 of the non-inoculated pigs, developed an infection (Hodson et al., 1975). Ransley and Risdon (1981) also developed a vesico-urethral reflux model using *E. coli* (Table 4). To sustain infection, the bacteria were inoculated within paraffin wax into the bladder. The aim of that study was to test different therapeutic methods for chronic pyelonephritis. The pigs developed marked interstitial renal fibrosis and were treated with different antibiotics without significant effect (Ransley and Risdon, 1981). In another study by Farhat et al. (2002), *E. coli* was also embedded in paraffin wax in order to sustain the infection. In the model, a total of 67% of the pigs developed renal scarring (Table 4; Farhat et al., 2002). Finally, in a novel model of acute pyelonephritis, a catheter was placed directly into the renal pelvis for inoculation (Table 4; Isling et al., 2011). Three different strains of *E. coli* with different virulence factors were used for inoculation (Isling et al., 2011). In the study, the strain which was positive for P fimbriae, an important virulence factor in the development of human pyelonephritis, developed the most pronounced lesions and mimicked the lesions seen in humans (Figures 3C2,C3; Isling et al., 2011).

**Table 4:** Porcine models of pyelonephritis.

**References (year)**	**Model design**							**Inoculum**				**Outcome**		
	**Number of animals**	**Age (weeks)**	**Weight (kg)**	**Sex**	**Breed**	**Infection time**	**Implant/modification**	**Bacteria**	**Dose CFU/ml**	**Volume**	**Route**	**Bacterial verification T/I**	**Infection stage**	Success rate^*^
Hodson et al., 1975	35	NR	NR	F	Sinclair mini-pig	7–130 days	Vesico-urethral reflux	*E. coli* 0111:B4	NR	NR	Intravesical	T: Microbiology + Histology I: NRV	Acute + Chronic	64%
Ransley and Risdon, 1981	43	2-4	NR	M/F	NR	1–4 weeks	Vesico-urethral reflux Paraffin wax	*E. coli* (strain NR)	NR	5–10 ml	Intravesical	T: Microbiology + Histology I: NR	Chronic	Control: 33–68% Gentamicin+ Chloramphenicol: 11% Nitrofurantoin: 19%
Farhat et al., 2002	10	3-4	10	F	Yorkshire	6–8 weeks	Vesico-urethral reflux Paraffin wax	*E. coli* (strain NR)	NR	NR	Intravesical	T: Microbiology + Histology + Ultrasound I: NR	Chronic	67%
Isling et al., 2011	9	NR	19	F	Yorkshire crossbred	6 h	Catheter	*E. coli* LK67, LK76 and LK82	10^9^	3.25 ml × 3	In renal pelvis	T: Microbiology + Histology I: NR	Acute	All strains: 88%

* *xThe percentage of animals with infection. T, verification method in tissue; I, verification method on implant; CFU, colony forming units; NR, not registered; F, female; M, male; NRV, not relevant*.

## Porcine models of hematogenous osteomyelitis

The rate of bone remodeling as well as the cross-sectional diameter of the femoral bone is similar in pigs and humans (Pearce et al., 2007). Pigs also show similarities to human bone composition, especially with respect to mineral density and mineralization of the bones (Aerssens et al., 1998). The muscles and bones of pigs are more massive compared to humans which reflects that they are quadruped (Swindle and Smith, 1998). Although, pigs have a denser osseous trabecular network, the lamellar bone structure is similar to humans (Pearce et al., 2007). Due to the similarities in bone composition and regeneration, the pig has been used as a model for several studies of both hematogenous and IAO (Wood et al., 1971; Koschmieder et al., 1975; Patterson et al., 1993; Rink et al., 2001; Jensen et al., 2010b, 2016,2017; Johansen et al., 2010, 2012a,b, 2013; Tøttrup et al., 2016).

In children, osteomyelitis is predominantly caused by hematogenous spread of bacteria. The lesions are most often located in long bones especially within the metaphysis of femur and tibia (Lew and Waldvogel, 2004). In adults, the vertebral bones are most commonly infected (Lew and Waldvogel, 2004; Brady et al., 2008). Following infection, an abscess will be formed, with a fibrous layer surrounding leukocytes and bacteria (Damjanov and Linder, 1996). The most common bacterium causing human osteomyelitis is *S. aureus*, but *S. epidermidis* is also often involved. Other bacteria involved in hematogenous osteomyelitis are *Streptococcus, Pneumococcus, E. coli, Klebsiella, Salmonella*, and *Bacteroides* (Damjanov and Linder, 1996). In slaughter pigs, the dominant site of infection is also within the metaphysis of long bones and the vertebrae (Figure 3D1). In pigs, hematogenous osteomyelitis is often caused by *T. pyogenes*, but *S. aureus* may also be the cause (Zachary, 2017). Although the same histomorphology is present, there will often be multifocal lesions in pigs (Maxie, 2007). In conventional pigs, the portal of entrance is often tail bites causing pyemia (Bækbo et al., 2016).

Several porcine models of hematogenous osteomyelitis have been developed. The first was established in 1971 by Wood et al. In that study, two different strains of *S. pyogenes* were inoculated through vena cava cranialis and they were inoculated once a day for three consecutive days (Table 5; Wood et al., 1971). The pigs developed endocarditis, arthritis and osteomyelitis. Some of the osteomyelitis lesions were caused by extension of the arthritis lesions and some were due to a direct hematogenous spread (Wood et al., 1971). In another study, pigs were inoculated intravenously with *S. aureus* and they developed microscopic metaphyseal osteomyelitis lesions after 12 h (Table 5; Jensen et al., 2010b). In a study by Johansen et al. (2010), *S. aureus* was inoculated into the brachial artery, in order to induce osteomyelitis in the distal part of the forelimb (Table 5; Johansen et al., 2010). It was found that the minimum required dose for inoculation was 5 × 10^3^ CFU/ml, in order to induce suppurative bone lesions (Johansen et al., 2010). In three other studies by Johansen et al. (2012a,b, 2013), *S. aureus* was inoculated into the femoral artery (Table 5; Johansen et al., 2012a,b, 2013). In the first study, three different strains of *S. aureus* were examined. The porcine strain, S54F9, resulted in the development of osteomyelitis lesions in all pigs. It was also shown that biofilm was present in the infected bone by PNA FISH (Johansen et al., 2012a). The novel technique of intraarterial inoculation used for inducing experimental osteomyelitis, was detailed in the second study by Johansen et al. (2012b; Figures 3D2,D3). In the third study from 2013, surgical debridement of experimental osteomyelitis was performed, and the lesions seen in the pigs were comparable to osteomyelitis lesions in kids. This study showed that the pig is a good model for evaluating surgical treatment methods for hematogenous osteomyelitis (Johansen et al., 2013).

**Table 5:** Porcine models of hematogenous osteomyelitis.

**References (year)**	**Model design**							**Inoculum**				**Outcome**		
	**Number of animals**	**Age (weeks)**	**Weight (kg)**	**Sex**	**Breed**	**Infection time**	**Implant/modification**	**Bacteria**	**Dose CFU/ml**	**Volume**	**Route**	**Bacterial verification T/I**	**Infection stage**	Success rate^*^
Wood et al., 1971	18	10–12	NR	M/F	Crossbred	58–63 days	None	*S. pyogenes* Strain Richards (type 3) and strain 25 (type 25)	Richards: 6.39 × 10^8^ Strain 25: 1.55 × 10^8^	3 ml	I.V.	T: Microbiology + Histology I: NRV	Chronic	Both strains: 83%
Jensen et al., 2010b	16	8	20-25	F	Yorkshire-Landrace crossbred	6–48 h	None	*S. aureus* Strain S54F9	1 × 10^8^	1 ml/kg (1–2x)	I.V.	T: Histology I: NRV	Acute	75%
Johansen et al., 2010	12	8–9	15	F	Yorkshire-Landrace crossbred	5–15 days	None	*S. aureus* Strain S54F9	5.50, 500, 5 × 10^3^ or 5 × 10^4^	0.5 ml	I.A.	T: Microbiology + Histology I: NRV	Acute	5 × 10^3^ CFU: 50% 5 × 10^4^ CFU: 100%
Johansen et al., 2012a	11	12	30	F	Yorkshire-Landrace crossbred	11–15 days	None	*S. aureus* Strain S54F9, NCTC-8325-4 and UAMS-1	10^4^	1 ml	I.A.	T: Microbiology + Histology (PNA FISH) I: NRV	Chronic	All strains: 44% S54F9: 100%
Johansen et al., 2012b	5	12	30	F	NR	11–15 days	None	*S. aureus* Strain S54F9	10^4^	1 ml	I.A.	T: Microbiology + Histology I: NRV	NR	100%
Johansen et al., 2013	4	12	30	M/F	Yorkshire-Landrace crossbred	6–8 days	None	*S. aureus* Strain S54F9	5 × 10^5^ and 5 × 10^6^	1 ml	I.A.	T: Microbiology + Histology I: NRV	Chronic	100%

* *The percentage of animals with infection. T, verification method in tissue; I, verification method on implant; CFU, colony forming units; NR, not registered; F, female; M, male; I.V., intravenous; I.A., intraarterial; NRV, not relevant*.

## Porcine models of implant-associated osteomyelitis

IAO is generally split into three groups depending on infection time following insertion of the implant, i.e., early, delayed, and late (Zimmerli et al., 2004). In humans, the most common type of infection is in the delayed group (3–24 months after surgery) and it is caused by local contamination by *S. aureus* or other bacteria during insertion of the implant (Figure 3E1; Zimmerli et al., 2004). The late type is caused by a hematogenous spread of bacteria colonizing the implant/prosthesis up to several years after insertion (Zimmerli et al., 2004). Histologically, a periprosthetic membrane surrounding the implant will be formed. This membrane can be divided into four different types (I–IV), where “Type II” is the infectious type. The infectious type is characterized by the proliferation of fibroblasts and small blood vessels, edema, and leukocyte infiltration, dominated by PMNs (Morawietz et al., 2006). Although, IAO does not occur spontaneously in pigs, it is assumed, that the pig will be a good model, due to its similarity in bone composition and remodeling (Pearce et al., 2007).

In a porcine model by Koschmieder et al. (1975), the effect of Gentamicin embedded in Palacos bone cement was examined (Koschmieder et al., 1975). In the study, IAO was established by traumatic intramedullary inoculation of *S. aureus* (Table 5), however, bacterial contamination was found and limited the conclusions of the study (Koschmieder et al., 1975). A traumatic mandibular IAO model was established in pigs using an 8 mm trephine (Table 5). In the model, three strains of *S. aureus* were inoculated intramedullary and afterwards, the trephine hole was filled with either bone cement or wax (Patterson et al., 1993). Another porcine IAO model was established by Rink et al. (2001) where an 18G needle was inserted in a mid-diaphysis fracture line and *S. aureus* was inoculated (Table 6). This was done in order to make a cDNA library of the cellular immune response in a porcine model of IAO (Rink et al., 2001) Recently, a novel porcine model of IAO was developed and comprehensively analyzed according to the local, regional and systemic response. In this model, a small Kirschner wire was inserted into the right tibial bone. Before the implant insertion, three different doses of *S. aureus* were applied (Table 6), and the model showed good reproducibility when an inoculum dose of 10^4^ CFU/ml was used (Figures 3E2,E3; Jensen et al., 2017). In a later study by Jensen et al. (2016), the former porcine IAO model was used to examine the extension of infection into the peri-implanted bone tissue after 5 days (Table 6; Jensen et al., 2016). Recently, a study performed by Tøttrup et al. (2016), focused on the penetration of cefuroxime into the infected bone lesions also using the porcine IAO model (Table 6; Tøttrup et al., 2016). The models of IAO mentioned above were all established successfully, and showed advantages for analyzing the bioavailability of antibiotics in e.g., bone cement or given systemically and the impact of the immune system. Moreover, as it also is the situation in human clinical settings, one should be aware of contamination problems when inserting implants.

**Table 6:** Porcine models of implant-associated osteomyelitis.

**References (year)**	**Model design**							**Inoculum**				**Outcome**		
	**Number of animals**	**Age (weeks)**	**Weight (kg)**	**Sex**	**Breed**	**Infection time**	**Implant/modification**	**Bacteria**	**Dose CFU/ml**	**Volume**	**Route**	**Bacterial verification T/I**	**Infection stage**	Success rate^*^
Koschmieder et al., 1975	5	12	NR	NR	Domestic landrace	16 days	Traumatic Bone cement	*S. aureus haemolyticus* (Strain NR)	2 × 10^8^	2 ml	Traumatic	T: Microbiology I: NR	NR	NR
Patterson et al., 1993	8	104–260	68–95	F	Yucatan mini-pigs	12 weeks	Traumatic Bone cement or bone wax	*S. aureus* ATCC strains 6538P, 25923, and 29213	10^8^–10^9^	1 ml	Traumatic	T: Microbiology I: NR	Chronic	All strains: 100%
Rink et al., 2001	10	NR	50-65	M	Yorkshire-Pietrain crossbred	28 days	18G needle	*S. aureus*	1.2 × 10^3^	NA	Traumatic	T: Radiography I: NR	NR	NR
Jensen et al., 2017	42	12 and 32	30 and 60–67	F	Danish landrace	5 days	Kirschner wire	*S. aureus* Strain S54F9 spa-type t1333	10^2^–10^4^	10 μl	Traumatic	T: Microbiology + Histology I: PNA FISH	Chronic	33- 83%
Jensen et al., 2016	12	NR	30	M/F	NR	2–6 days	Kirschner wire	*S. aureus* Strain S54F9 spa-type t1333	10^4^	10 μl	Traumatic	T: Microbiology + Histology I: PNA FISH + SEM	Acute + Chronic	67%
Tøttrup et al., 2016	10	NR	67–77	F	Danish landrace	5 days	Kirschner wire	*S. aureus* Strain S54F9 spa-type t1333	10^4^	10 μl	Traumatic	T: Microbiology + Histology I: NR	Acute	100%

* *The percentage of animals with infection. T, verification method in tissue; I, verification method on implant; CFU, colony forming units; NR, not registered; F, female; M, male*.

## Summary

The basic knowledge of infectious biofilm has been achieved from *in vitro* studies (Lebeaux et al., 2013). However, biofilm grown *in vitro* is not in every respect comparable to biofilm produced *in vivo* (Bjarnsholt et al., 2013). Therefore, it is advantageous to perform studies of infectious biofilm using *in vivo* experiment, as this will allow studies of the host response with regard to the mechanisms of formation, immune response and therapeutically (Coenye and Nelis, 2010; Lebeaux et al., 2013; Stewart, 2014).

The pig has proven to be an appropriate animal model for the study of chronic bacterial biofilm diseases (Sullivan et al., 2001). The pig allows for several therapeutical trials and also shares a number of similarities to humans with respect to anatomy and the immune system (Swindle and Smith, 1998; Dawson, 2011; Meurens et al., 2012; Swindle et al., 2012). Although the present review highlights the many advantages of using pigs for modeling of human bacterial infectious diseases, small animal models are still used more extensively for the same purposes (Reizner et al., 2014). Common reported advantages of small animal models are related to cost, housing, and handling (Reizner et al., 2014). However, when applying an animal model one of the most important issues should be that the model is discriminative for the disease seen in humans. Due to the general increase in the use of pigs as experimental animals and an increased awareness of reliability in animal models, more porcine models of human bacterial infections are expected to be developed in the future.

Another important factor, which makes the pig a preferable model for many chronic infectious diseases, is that pigs develop many of the diseases spontaneously (Anderson, 1976; Damjanov and Linder, 1996; Maxie, 2007; Jensen, 2009; Jensen et al., 2010a). The pathogenesis and lesions of spontaneously occurring chronic pressure wounds, endocarditis, pyelonephritis, and hematogenous osteomyelitis are similar to those seen in humans and are also often caused by the same bacteria (Anderson, 1976; Damjanov and Linder, 1996; Maxie, 2007; Jensen, 2009; Jensen et al., 2010a). Although IAO does not occur naturally in pigs, porcine models have proven useful as a model because the induced lesions show great similarities to those of humans (Jensen et al., 2017).

When developing a chronic bacterial infectious animal model, it is important to validate whether or not biofilm is formed. Biofilm can be formed both in tissue and on implants, therefore, in future studies it is important to analyze both (Donlan and Costerton, 2002). Biofilm is an important factor in sustaining chronic infections in humans (Donlan and Costerton, 2002; Costerton et al., 2003; Burmølle et al., 2010). The ability of bacteria to form biofilm in porcine models has been demonstrated in a number of studies (Davis et al., 2007, 2013; Johansen et al., 2012a; Roche et al., 2012; Jensen et al., 2016). However, when looking at the studies in this review (Tables 2–6), only five out of the thirty studies, actually commend on the formation of biofilm (Davis et al., 2007, 2013; Johansen et al., 2012a; Roche et al., 2012; Jensen et al., 2016). This probably reflects that most of the studies were carried out decades ago and biofilm is a new focus of interest in regard to chronic infectious diseases. This may also be the reason why biofilm is usually not mentioned in chronic, spontaneous porcine infections (Maxie, 2007). However, as seen from the descriptions of infections in the present review (Figure 3) it is likely that all chronic porcine infections are the cause of biofilm formation as in humans (Donlan and Costerton, 2002; Costerton et al., 2003; Brady et al., 2008). This assumption has also been supported in porcine pneumonia due to *Actinobacillus pleuropnumoniae* (Tremblay et al., 2017).

In the future, more models of chronic bacterial biofilm infections should be examined in pigs. Among others, studies of cystic fibrosis and otitis media based on porcine models would be relevant, as the pig has proven to have great similarity to humans with respect to anatomy of the respiratory tract and the conformation of the middle and inner ear (Pracy et al., 1998; Meurens et al., 2012; Schomberg et al., 2016).

## Author contributions

LJ and HJ designed the structure of the review. AJ collected all the references. LJ, AJ, and HJ drafted the manuscript.

### Conflict of interest statement

The authors declare that the research was conducted in the absence of any commercial or financial relationships that could be construed as a potential conflict of interest.
